# OGG1 competitive inhibitors show important off-target effects by directly inhibiting efflux pumps and disturbing mitotic progression

**DOI:** 10.3389/fcell.2023.1124960

**Published:** 2023-02-03

**Authors:** Xhaferr Tanushi, Guillaume Pinna, Marie Vandamme, Capucine Siberchicot, Ostiane D’Augustin, Anne-Marie Di Guilmi, J. Pablo Radicella, Bertrand Castaing, Rebecca Smith, Sebastien Huet, François Leteurtre, Anna Campalans

**Affiliations:** ^1^ Université Paris-Saclay, CEA/IBFJ/IRCM. UMR Stabilité Génétique Cellules Souches et Radiations, Fontenay-aux-Roses, France; ^2^ Université de Paris-Cité, CEA/IBFJ/IRCM. UMR Stabilité Génétique Cellules Souches et Radiations, Fontenay-aux-Roses, France; ^3^ Université Paris-Saclay, Inserm, CEA/IBFJ/IRCM/Plateforme PARi, UMR Stabilité Génétique Cellules Souches et Radiations, Fontenay-aux-Roses, France; ^4^ Université de Paris-Cite, Inserm, CEA/IBFJ/IRCM/Plateforme PARi, UMR Stabilité Génétique Cellules Souches et Radiations, Fontenay-aux-Roses, France; ^5^ Université Rennes, CNRS, IGDR (Institut de Génétique et Développement de Rennes)—UMR 6290, BIOSIT—UMS 3480, Rennes, France; ^6^ Centre de Biophysique Moléculaire (CBM) UPR4301 CNRS, Université d’Orléans, Orléans, France; ^7^ Institut Universitaire de France, Paris, France

**Keywords:** Base Excision Repair (BER), OGG1, 8-oxoG, TH5487, SU0268, OGG1 inhibitor

## Abstract

One of the most abundant DNA lesions induced by Reactive oxygen species (ROS) is 8-oxoG, a highly mutagenic lesion that compromises genetic instability when not efficiently repaired. 8-oxoG is specifically recognized by the DNA-glycosylase OGG1 that excises the base and initiates the Base Excision Repair pathway (BER). Furthermore, OGG1 has not only a major role in DNA repair but it is also involved in transcriptional regulation. Cancer cells are particularly exposed to ROS, thus challenging their capacity to process oxidative DNA damage has been proposed as a promising therapeutic strategy for cancer treatment. Two competitive inhibitors of OGG1 (OGG1i) have been identified, TH5487 and SU0268, which bind to the OGG1 catalytic pocket preventing its fixation to the DNA. Early studies with these inhibitors show an enhanced cellular sensitivity to cytotoxic drugs and a reduction in the inflammatory response. Our study uncovers two unreported off-targets effects of these OGG1i that are independent of OGG1. *In vitro* and *in cellulo* approaches have unveiled that OGG1i TH5487 and SU0268, despite an unrelated molecular structure, are able to inhibit some members of the ABC family transporters, in particular ABC B1 (MDR1) and ABC G2 (BCRP). The inhibition of these efflux pumps by OGG1 inhibitors results in a higher intra-cellular accumulation of various fluorescent probes and drugs, and largely contributes to the enhanced cytotoxicity observed when the inhibitors are combined with cytotoxic agents. Furthermore, we found that SU0268 has an OGG1-independent anti-mitotic activity—by interfering with metaphase completion—resulting in a high cellular toxicity. These two off-target activities are observed at concentrations of OGG1i that are normally used for *in vivo* studies. It is thus critical to consider these previously unreported non-specific effects when interpreting studies using TH5487 and SU0268 in the context of OGG1 inhibition. Additionally, our work highlights the persistent need for new specific inhibitors of the enzymatic activity of OGG1.

## Introduction

Base excision repair (BER) copes with oxidized, alkylated and deaminated DNA damaged bases in a four steps mechanism: 1) recognition and cleavage of damaged base by one of the 11 DNA glycosylases, 2) incision of the resulting abasic site, 3) end processing and 4) repair synthesis trough gap filling and ligation. Among all, one of the most abundant base modifications induced by oxidative stress is 8-oxoG, that is specifically recognized and repaired by the DNA glycosylase OGG1, a protein playing a major role on the stability of both nuclear and mitochondrial genomes (d’Augustin et al., 2020; Lebraud et al., 2020; Lia et al., 2018; Boiteux et al., 2017). If unrepaired, 8-oxoG leads to G:C → T:A transversions, a frequent and diffuse somatic mutational signature in cancer genomes (Greenman et al., 2007), that is thought to be responsible of both cancer onset and progression. On the other hand, excessive glycosylase activity following acute DNA damage is detrimental for genomic stability because of the toxicity of repair-intermediates, suggesting the need to balance BER (Dianov and Hübscher, 2013). Beside its mutational and genome instability potential, an increasing body of evidence has pointed at 8-oxoG as an epigenetic marker through which OGG1, with or without its catalytic activity, can either activate or repress the transcription of different cancer relevant genes (Hahm et al., 2022).

Taken together, the above-mentioned features of the 8-oxoG-OGG1 pair lead to the idea that modulating OGG1 catalytic activity may be a promising intervention for cancer treatment. Therefore, a lot of efforts have been made to identify small-molecules able to either inhibit (Donley et al., 2015; Visnes et al., 2018a; Tahara et al., 2018) or enhance (Michel et al., 2022; Tian et al., 2022) OGG1 catalytic activity. OGG1 inhibitors were identified by screening libraries composed of several thousand of small chemical compounds that were evaluated for their ability to inhibit the *in vitro* DNA glycosylase activity of purified OGG1. Two of the identified OGG1 inhibitors, SU0268 (Tahara et al., 2018) and TH5487 (Visnes et al., 2018a) act as competitor inhibitors by taking the place of the 8-oxoG in the active site of the enzyme and thus impairing the recognition and repair of genomic 8-oxoG.

OGG1 inhibitors have been shown to dampen cytokines expression in cancer cells and other models, therefore reducing inflammatory responses (Visnes et al., 2018a; Xia et al., 2022; Hajnády et al., 2022), specifically arrest cancer cell proliferation (Visnes et al., 2020) and to enhance methotrexate effect on cancer cells through telomeres instability (Baquero et al., 2021). OGG1 inhibitors are therefore proposed as potential promising therapeutical tools for the treatment of cancer (Visnes et al., 2018b).

However, before moving to clinics the molecular mechanisms of OGG1 inhibitors should be carefully evaluated. One of the major problems of chemical inhibitors is their off-target effects. Indeed a recent study has shown that most of the chemical drugs identified up to now kill cancer cells independently of their supposed target (Lin et al., 2019). Consequently, medical agencies, including the Food and Drug Administration (FDA) recommend investigation of a drug candidate with regard to several parameters, including its effect on ABC (ATP Binding Cassette) transporters.

The ATP-binding cassette (ABC) transport superfamily consists of 48 proteins organised in seven subfamilies (A–G). These proteins are localized at the plasma-membrane but are also found in the organelle membranes and are widely expressed across human cell-types and tissues (Wang et al., 2021). They are responsible of the transport of a plethora of exogenous and endogenous molecules in a unidirectionally manner, feature that owes them also the name of “efflux pump”.

High expression levels and mutations of 13 ABC proteins have been independently associated with poor prognosis in different cancer types (Kadioglu et al., 2020). Among these, the most important and better characterized are: ABCB1/MDR1 (multidrug resistance 1), ABCC1/MRP1 (multidrug resistance-associated protein 1) and ABCG2/BCRP (Breast-cancer resistance protein). Their overexpression, alone or in a combinatory way (Marzac et al., 2011), confers cancer cells multiple-drug resistance through the efflux of different chemotherapeutics due to poor substrate specificity and high redundance between pumps. This mechanism is specifically exploited by cancer stem cells (CSCs), whose resistance to treatments is at the base of cancer recurrence (Moitra et al., 2011). However, ABC transporter’s role is not limited to chemotherapeutics efflux, they also play a role in a variety of cancer hallmarks such as enhanced proliferation (Bhattacharya et al., 2007; Katoh, et al., 2008), resistance to cell death (Smyth et al., 1998; Tainton et al., 2004), migration and invasion (Miletti-González et al., 2005; Colone et al., 2008). ABC transporters are also involved in the shuttling of lipids, prostaglandins and leukotriens and are thus involved in metabolic reprogramming, angiogenesis and formation of a pro-inflammatory tumour microenvironment (Fletcher et al., 2010).

These features have prompted the scientific community to target ABC transporters in cancer to reverse multidrug resistance. To date, all the clinical trials have failed because of severe side-effects and poor responses. While the safety and specificity has been optimized in the latest inhibitors generation, the multiple functions (Chen et al., 2020) of ABC transporters are remaining challenges for precise medicine intervention and urge to be revisited (Robey et al., 2018).

In the present work, we identified two out of three tested cancer associated ABC transporters, ABCB1/MDR1 and ABCG2/BCRP, as off-targets of the OGG1 inhibitors TH5487 and SU0268. Furthermore, we have also observed an off-target effect of SU0268 on mitotic progression. We discuss here how this off-target effects may influence the interpretation of the results obtained upon exposure of the cells to OGG1 inhibitors and also which impact this could have for the therapeutical use of this molecules in cancer treatment.

## Material and methods

### Cell culture and treatments

U2OS cells were routinely maintained in DMEM (21885025, Thermo Fisher) supplemented with 10% (v/v) Fetal Bovine Serum (PAA), Penicillin (100 U/ml) and Streptomycin (100 µg/ml). A549 and SH-SY5Y were grown in DMEM (31966021, Thermo Fisher) while HMLE-E have been maintained in 1:1 Dulbecco’s Modified Eagle Medium (DMEM)/HAMF12 medium supplemented with 10% fetal calf serum, penicillin, streptomycin, amphotericin B (Life Technologies), 10 ng/mL human epidermal growth factor (EGF) (Sigma), 0.5 pg/mL hydrocortisone (Sigma) and 10 pg/mL insulin (Sigma).

For the generation of U2OS OGG1 knockout cell lines two target sequences were chosen (in exons 2 and 3), according to the web-based CRISPR design tool CRISPick (https://portals.broadinstitute.org/gppx/crispick/public). The sgRNA oligos targeting exon 2 (CAC​CGC​TCA​ACT​GTA​TCA​CCA​CTG) or exon 3 (CAC​CGA​AAG​AGA​AAA​GGC​ATT​CGA​T) were introduced into pX458 expressing Cas9 nuclease fused to GFP. pSpCas9 (BB)-2AGFP (PX458) was a gift from Feng Zhang (Addgene plasmid #48138). U2OS WT cells were transfected with the guide containing plasmid using the transfection reagent Lipofectamine 2000 (Thermo Fisher Scientific) according to manufacturer’s protocol. Single GFP positive cells were sorted into 96-well plates using the BD Influx sorter (BD Biosciences). The knockout cell lines grown from the single cells were screened by western blot using an antibody against OGG1 (Abcam, ab124741).

OGG1 inhibitors TH5487 (M9506) and SU0268 (HY-139056 (MedChemExpress); 10-4700 (Focus Biomolecules) and MBS5765223 (MyBiosource)) were purchased from Clinisciences. Efflux pump inhibitors Verapamil (V4629), Fumitremorgin C (344847) and Zosuquidar/LY-335979 (SML1044) were purchased from Merck. The dyes Picogreen (P7581), Mitotracker green (M7514) and TMRE (T669) were obtained from Thermo Fisher and SiR-DNA (SC007) from SpiroChrome. The drugs Nocodazole (M1404), Etoposide (E1383) and KBrO_3_ (309087) and Mitoxantrone (M6545) were purchased from Sigma.

### Cellular staining and imaging

For the quantification of γH2AX foci wild-type and OGG1-KO U2OS cells were resuspended in culture medium and seeded into an optical 96-well culture plate (89626, Ibidi) at a final density of 4000 cells/well. Plates were then incubated for 3 days at 37°C, 5% CO2. The day of the experiment, cells were treated with the indicated drugs at the indicated concentrations, and plates were returned at 37°C for 1 h. Supernatants were then removed and cells were fixed by adding 100 µL of a 4 % formaldehyde solution (w/v, 47608, Sigma) diluted in PBS with Ca2+/Mg2+ (D8662, Sigma). After 20 min, formaldehyde was removed and wells were washed three times with 200 µL PBS without Ca2+ & Mg2+ (D8537, Sigma). Cells were permeablized 10 min at RT with 150 μl of Triton-X100 (X100, Sigma) 0.5% (v/v) in PBS without Ca2+ & Mg2+. Wells were washed three times with 200 µl of PBS without Ca2+ & Mg2+, and non-specific epitopes were saturated for 60 min at RT with 150 µl of PBS + 2% BSA (w/v, A5611, Sigma) + Tween20 0.1% (v/v, P9416, Sigma). After blocking buffer removal, 100 µl of anti- γ-H2AX mouse antibody (JBW301 clone, 05-636-I, Sigma) diluted at 1:1000 in PBS + 1% BSA (w/v) + Tween 0.1% (v/v) were layered into the wells. Plates were incubated at room temperature for 90 min, then wells were washed three times with 200 µl of PBS without Ca2+ & Mg2+. 100 µl of Goat anti-Mouse antibody coupled to Alexa-488 diluted at 1:1000 in PBS + BSA 1% (w/v) + Tween 0.1% (v/v) were layered into the wells. Plates were incubated at room temperature for 90 min, then wells were washed three times with 200 µl of PBS without Ca2+ & Mg2+. Nuclear DNA was then probed 20 min with 150 µl of Hoechst 33342 (#B2261, Sigma) diluted at 2 μg/mL in PBS without Ca2+ & Mg2+. Wells were then washed two times with 200 µl of PBS without Ca2+ & Mg2+, and 200 µl of PBS were added to the wells. Plates were sealed with an aluminium foil and imaged on a high-content imaging microscope (Operetta, Perkin Elmer) at ×20 and ×40 magnification (13 fields acquired/well) in the blue (Ex. 360–400 nm, Em. 410–480 nm) and green (Ex. 460–490 nm, Em. 500–550 nm) channels. Using a high-content imaging analysis software (Harmony 3.0, Perkin-Elmer), nuclear ROI were segmented in the Hoechst (blue) channel and the number of γ-H2AX foci per nuclei was quantitated in ×40 magnification images using a foci-detection algorithm.

For visualization of 8-oxoG, HeLa OGG1–GFP cells grown on 8-well glass bottom chamber slides (80827, ibidi) were fixed in acetone:methanol (1:1) and air dried. Cells were hydrated for 15 min in PBS, and DNA was denatured by incubating cells in 2N HCl for 45 min at room temperature. Cells were washed three times in PBS, neutralized with 50 mM Tris–HCl pH 8.8 for 5 min and permeabilized at room temperature in PBS–0.1% Triton for 10 min. Cells were incubated in blocking solution (PBS, 0.1% Triton, 3% BSA, 1% normal goat serum) at 37°C for 1 h and subsequently incubated for 1 h at 37°C with antibodies against 8-oxoG (ab48508, abcam) and GFP (632592, Clontech) diluted in blocking solution at 1:2000 and 1:1000 respectively. Cells were then washed three times for 10 min in PBS–.1% Triton and incubated with an anti-Mouse antibody coupled to Alexa-488 (A11001, Thermofisher) and an anti-Rabbit antibody coupled to Alexa-647 (A31573, Thermofisher), both diluted at 1:1000 in blocking solution for 1 h at 37°C. Nuclear DNA was counterstained with 1 μg/ml propidium iodide with 50 μg/ml RNAse. Cells were imaged with a Nikon A1 confocal microscope, using a ×60 oil immersion objective with a numerical aperture of 1.3 and analysis was performed with Fiji (Schindelin et al., 2012).

For stainigs with Picogreen and Nuclight, wild-type and OGG1-KO U2OS cells were resuspended in culture medium and seeded into the wells of an optical 96-well culture plate (#3904, Costar) at a final density of 2000 cells/well. Plates were then incubated for 3 days at 37°C, 5% CO2. The day of the experiment, cells were first incubated 30 min at 37°C with Picogreen (1:500 [V/V] dilution from the supplier’s stock solution, Invitrogen). Supernatants were then removed and wells were washed once with 150 µL PBS with Ca2+/Mg2+. 50 µl of a suspension of complete medium containing Nuclight (1:500, v/v, #4717, Sartorius) alone or mixed together with the indicated inhibitors were layered into the culture wells. After 1 h of incubation at 37°C, supernatants were removed and replaced with 150 µl of complete medium. Plates were immediately imaged on a high-content imaging microscope (Operetta, Perkin Elmer) at ×10 magnification (9 fields acquired/well) in the green (Ex. 460–490 nm, Em. 500–550 nm) and far red (Ex. 620–640 nm, Em. 650–760 nm) channels. Using a high-content imaging analysis software (Harmony 3.0, Perkin-Elmer), Picogreen fluorescence was used to segment a nuclear Region of Interest (ROI), in which the average Nuclight fluorescence intensity was quantitated.

For DNA staining with Hoecsht 33342, cells grown on 8-well glass bottom chamber slides were incubated for 5 min at 37°C in medium containing the dye at 1 μg/ml, washed twice in warm medium and immediately imaged with a spinning disk microscope (GATACA, W1) using a ×60 oil objective and an excitation laser at 405 nm.

### Plate reader (CLARIOSTAR)

Cells were seeded at 10.000 cells/well in 96 well plates and 3 days after seeding cells were incubated for 1 h in DMEM containing either DMSO (NT), or the OGG1i TH5487 or SU0268 at a concentration of 10 µM. Cells were further stained for 20 min with 100 nM of TMRE and 100 nM of MitoTracker Green in the presence of OGG1i and fluorescence was immediately quantified using a microplate reader (CLARIOstar—BMG Labtech).

### Flow cytometry analysis

Cells were trypsinized and resuspended to a final concentration of 2 million cells/mL. For each condition, 400′000 cells (200 µl) were distributed in flow cytometry tubes and OGG1i or pump inhibiting drugs were added at a 2X of final concentration. On top of that, 200 µl of warm medium containing the investigated dye at 2X of final concentration (MitoTracker Green 100nM, TMRE 100nM, Sir-DNA 333nM, Hoechst33342 0.1 μg/μl, and Mitoxantrone 1 µM) was added. Cells were incubated 30 min at 37°C and further 10 min at room temperature for equilibration before flow cytometry analysis with the NovoCyte Flow Cytometer System (Agilent).

The immunostaining for Phospho-H3 was performed in cells resuspended, fixed and permeabilized in ice-cold 75% ethanol. Upon washing, cells were blocked in PBS buffer containing 1% BSA, 0.05% Triton X100 and cells were incubated overnight at 4°C with an Pacific Blue Conjugate monoclonal antibody against Phospho-Histone H3 (Ser10) (8552, Cell Signaling) diluted at 1:500 in PBS buffer containing 1% BSA, 0.05% Triton X100. Washed cells were resuspended in PBS-1% BSA in the presence of 0.3 µM of Topro-3 for DNA staining 1 h at room temperature before FACS analysis with the NovoCyte Flow Cytometer System (Agilent).

### Western blots

Cell pellets (from 3 × 10exp6 cells) were resuspended in 50 µl of Lysis buffer (Tris 20 mM, NaCl 20 mM, SDS 0.1%, MgCl_2_ 1 Mm, Benzonase 0.25 U/µl and Protease inhibitors cocktail 1X) and sonicated for 10 min (with pulses 30 s on/30 s off). After sonication the samples were centrifuged at 12000 at 4°C for 5 min and the supernatants were recovered. Protein concentration was measured with the Bradford assay (Bio-Rad #500-0006), laemmli buffer was added at a 1x concentration (0.1% 2-mercaptoethanol, 0.0005% Bromophenol blue, 10% glycerol, 2% SDS, and 63 mM Tris pH 6.8) before heating for 5 min at 95°C, and 20 µg of protein extracts were loaded in a mini PROTEAN TGX stain-free gel (Bio-Rad #4568083). Precision plus protein dual color standards (Bio-Rad #1610374) was used as a Molecular weight marker. The transfer on nitrocellulose membrane (Bio-Rad #1704157) was performed with the Trans-Blot^®^ Turbo™ Transfer System (Bio-Rad #1704150). The membrane was blocked for 1 h in blocking solution (PBS-0.1% Tween20 containing 5% milk), and incubated for 1 h at room temperature with primary antibodies (anti-OGG1 Ab124741 1:10000 and Anti-Vinculine Ab18058 1:4000). Membranes were washed three times for 5 min with PBS-0.1% Tween20 and further incubated for 45 min in secondary antibodies anti-rabbit IR800 (Diagomics R-05060) and anti-mouse IR700 (Diagomics R-05055) diluted at 1/10.000 in PBS-0.1% Tween 20 containing 5% milk. Western blots were imaged with the Li-Cor Odyssey DLx system.

### RNA extraction and RT-qPCR

RNA was extracted from 1 × 10 exp6 cells using RNeasy Plus micro kit (#74034, QIAGEN) following manufacturer instructions and the concentration measured with Nanodrop 2000c (Thermo Fisher Scientific). 300 ng of RNA was used for the reverse transcription with the Thermo-Fisher Super Script Vilo kit (#11754050) using the following cycle: 1 min at 25°C, 60 sec at 42°C and 5 sec at 85°C in Eppendorf thermocycler (Eppendrof Mastercycler ep gradient S). Resulting cDNAs were amplified by qPCR with the iTaq Universal Probes Supermix (Biorad #1725130, Biorad) using the Applied Biosystems™ QuantStudio™ three Real-Time PCR System. Taqman probes against MDR1 (Hs00184500_m1), BCRP1 (Hs01053790_m1), and GAPDH (HS9999905-m1) were purchased from Thermo Fisher Scientific.

### Vesicular transport inhibition assay

Vesicular transport inhibition assays were carried out by SOLVO^®^ Biotechnology. Briefly, vesicles from HEK293 cells overexpressing MDR1, BCRP or MRP1 were purified to have an inside-out pump configuration. The reaction mix containing the vesicles, the specific corresponding radiolabeled substrate [N-methyl-quinidine (NMQ), Estrone-3-sulfate (E3S) or β-estradiol-17-β-D-glucuronide (E217β)], and specific corresponding pump inhibitor [Valspodar (1 μM), Ko143 (0.2 μM) or Benzbromarone (200 μM)), OGG1i (TH5487 and SU0286 (10 and 100 μM)] or DMSO were resuspended in transport buffer and incubated for 15 min at 37°C for MRP1 and 32°C for MDR1 and BCRP. The start reagent solution, containing a final concentration of either 4 mM Mg-ATP or 4 mM AMP in transport buffer, was added on top of the reaction mix. The reactions were incubated for 1 min for BCRP and MDR1 and for 3 min for MRP1 and quenched by the addition of ice-cold washing mix. The samples were transferred to a filter plate and washed 5-times with ice-cold washing mix, and the amount of probe substrate inside the filtered vesicles was quantified by liquid scintillation counting and expressed as counts per million (cpm). For each studied condition, the cpm values in the presence of ATP were subtracted by the corresponding values in the presence of AMP (passive diffusion of the substrate inside the vescicles, background signal). The resulting values have been normalized to DMSO and expressed in % as a measure of pump inhibition effect for each tested drug.

### Cell proliferation

For the proliferation curves, 1000 cells resuspended in 75 µl culture complete media were seeded in a 96 well plate. After 6 h, 25 µl of culture medium containing or not OGG1i at 4X of final concentration were added. Cell proliferation was followed for 1 week with the Incucyte^®^ S3 System by acquiring five pictures per well at an image rate of one image every 3 h. The resulting transmission images were analysed with the Incucyte^®^ S3 segmentation algorithm generating a coverage mask used to follow cellular confluence.

To evaluate cellular sensitivity to Etoposide treatments, wild-type and OGG1-KO U2OS cells were resuspended in culture medium and seeded into the wells of an optical 96-well culture plate (#3904, Costar) at a final density of 1000 cells/well. Plates were incubated 24 h at 37°C, 5% CO2 to allow for cell adhesion. 24 h post-seeding, cells were treated with the indicated drugs at the indicated concentrations for 1 h at 37°C, washed with fresh medium and incubated for 96 h at 37°C, 5% CO2. At the end of the incubation period, cells were fixed with formaldehyde 4% solution, diluted in PBS with Ca2+/Mg2+ containing Hoechst 33342 at 2 μg/mL. After an overnight incubation at 4°C in the dark, fixation solution was removed by aspiration, replaced by 150 µl of PBS with Ca2+ and Mg2+, and plates were imaged on a high-content imaging microscope (Operetta, Perkin Elmer) at ×10 magnification (9 fields acquired/well) in the blue channel (Ex. 360–400 nm, Em. 410–480 nm). Using a high-content imaging analysis software (Harmony 3.0, Perkin-Elmer), DNA-labeled nuclei were segmented, and the absolute amount of nuclei per condition was quantitated. Results were expressed as the amount of cells relative to the average amount of cells in the untreated wells.

### Statistical analysis

Statistical analysis were performed with FlowJo or GraphPad Prism 9.1.2 softwares. The statistical test used is indicated in the Legend to the Figures.

## Results

### Staining with both nuclear and mitochondrial fluorescent markers is improved in the presence of OGG1 inhibitors TH5487 and SU0268, in an OGG1 independent manner

**FIGURE 1:**
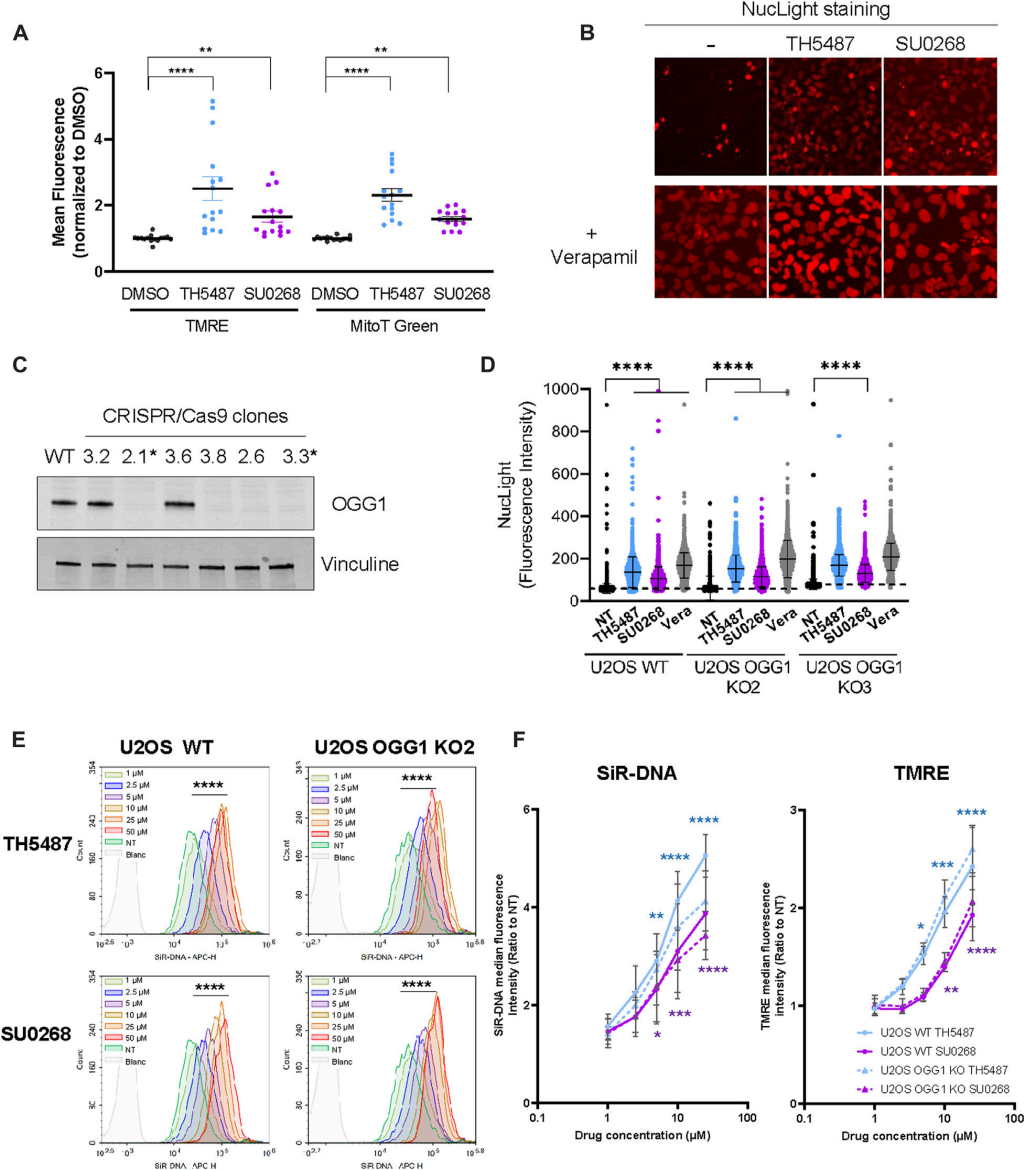
The addition of OGG1 competitive inhibitors TH5487 and SU0268 to the medium results in higher staining with fluorescent dyes. **(A)** U2OS cells were stained with mitochondrial markers MitoTracker Green and TMRE in the presence of 10 µM of OGG1 inhibitors TH5487 or SU0268. Control cells were stained in DMEM containing DMSO at .1%. Fluorescence was acquired in a plate reader. Fluorescent intensity for each of the channels was normalized to the values measured in cells stained in control cells. Ploted values correspond to at least three technical replicates from four independent experiments. Statistical analysis was performed with a Kruskal-Wallis test. (****)*p* < 0.0001; (**) *p* < 0.005. **(B)** U2OS cells were stained with NucLight in the presence or absence of OGG1 inhibitors TH5487 and SU0268 combined or not with Verapamil **(A)** and imaged with the Incucyte^®^ Live-Cell Analysis System. **(C)** OGG1 KO cells generated by CRISPR/Cas9 were validated by Western Blot analysis using an antibody against OGG1. Vinculine was used as a loading control. Clones 2.1 and 3.3 were selected and used in further experiments. **(D)** U2OS WT and OGG1 KO cells, were stained with NucLight and Picogreen in the presence or absence of OGG1 inhibitors or Verapamil (all used at 10 µM) and imaged with a high-content imaging microscope in both the green and the red channels. Representative images used for the quantification are presented in Figure S1. The graph corresponds to the NucLight fluorescence intensity measured in the nucleus for up to 7000 cells for each condition. One representative experiment out of three is presented. Statistical analysis was performed with a Kruskal-Wallis test. (****)*p* < 0.0001. **(E)** U2OS WT and OGG1 KO cells were stained with SiR-DNA in the presence of different concentrations of TH5487 and SU0268 (1, 2.5, 5, 10, 25 or 50 µM) and analysed by flow-cytometry. Results from a representative experiment out of three are presented. The fluorescence distributions of cells stained in the presence of different concentration of TH5487 or SU0268 were compared to control cells on FlowJo using a chi-squared test (****) *p*-value < 0.001). **(F)** U2OS WT and OGG1 KO cells (clone 2.1) were stained with SiR-DNA or TMRE in the presence of different concentrations of TH5487 and SU0268 (1, 2.5, 5, 10, 25 or 50 µM) and analysed by flow-cytometry. The median of at least three independent experiments is shown. Statistical analysis was performed with Ordinary one-way ANOVA (*)*p* < 0.05; (**)*p* < 0.01; (***)*p* < 0.001; (****)*p* < 0.0001.

In our aim to evaluate the impact of OGG1i in both nuclear and mitochondrial function, U2OS cells exposed or not to OGG1i were stained with the mitochondrial probes Mitotracker green and TMRE, indicators of mitochondrial mass and activity respectively. Surprisingly, the levels of fluorescence obtained were significantly higher when the staining was done in the presence of OGG1 inhibitors TH5487 and SU0268 (Figure 1A). Similar observations were made with the cell permeant DNA probes SiR-DNA and NucLight, classically used for nuclear staining in living cells. While NucLight very slightly stained the nucleus of U2OS, staining was again much stronger in the presence of the two OGG1 inhibitors (Figure 1B). It is known that those dyes can be exported by the efflux pumps and that’s the reason why they are often combined with the general efflux pump inhibitor Verapamil in order to improve cellular staining (Lukinavičius et al., 2015). As expected, the incubation of cells with NucLight in the presence of Verapamil resulted in a much stronger nuclear staining that was very similar to the one obtained with the OGG1 inhibitors SU0268 and TH5487. To better quantify the observed effects, cells were simultaneously stained with another DNA-intercalator, Picogreen, prior to the incubation with NucLight. Picogreen staining was used for the segmentation of the nucleus and the generation of a mask that was applied to the NucLight image in order to quantify the nuclear staining. A significant increase in the NucLight staining was observed in the presence of TH5487 and SU0268, again similar to the observed with the efflux pump inhibitor Verapamil (Figure 1D and Supplementary Figure S1). Exactly the same effects were observed in two independent U2OS cell lines in which OGG1 was knocked out by CRISPR-Cas9 using guideRNAs targeting exon 2 (KO2) or exon 3 (KO3) (Figures 1C, D). These results indicate that the observed effects of the DNA glycosylase inhibitors were not dependent on OGG1 but necessarily an off-target effect.

Dose response curves using different concentration of OGG1 inhibitors were performed by flow cytometry analysis using SiR-DNA (Figure 1E) and TMRE (Figure 1F) and clearly showed that the observed effects were dependent on the concentration of OGG1 inhibitors. Here again, the same observations were made in WT cells and in cells deficient for OGG1 confirming the off-target effect (Figures 1E, F).

Considering the unexpected effects of OGG1 inhibitors reported here, we decided to evaluate if those molecules were still acting as OGG1 inhibitors in our experimental conditions. With that purpose, we determined the repair kinetics of 8-oxoG following exposure of cells to KBrO3, a drug known to induce large amounts of 8-oxoG in genomic DNA. Cells overexpressing OGG1-GFP were used in this experiment in view of their faster repair kinetics compared to the cells expressing OGG1 at endogenous levels (Amouroux et al., 2010; Lebraud et al., 2020; Supplementary Figures S2A–C). As shown in Supplementary Figures S2C,D, repair of the 8-oxoG was clearly impaired in cells incubated in the presence of OGG1 inhibitors TH5487 and SU0268. Therefore, we concluded that despite the capacity of these molecules to inhibit the enzymatic activity of OGG1, they show off-target effects at the cellular level that are independent on OGG1.

### TH5487 and SU0268 directly inhibit efflux pumps BCRP (ABCG2) and MDR1 (ABCB1)

**FIGURE 2:**
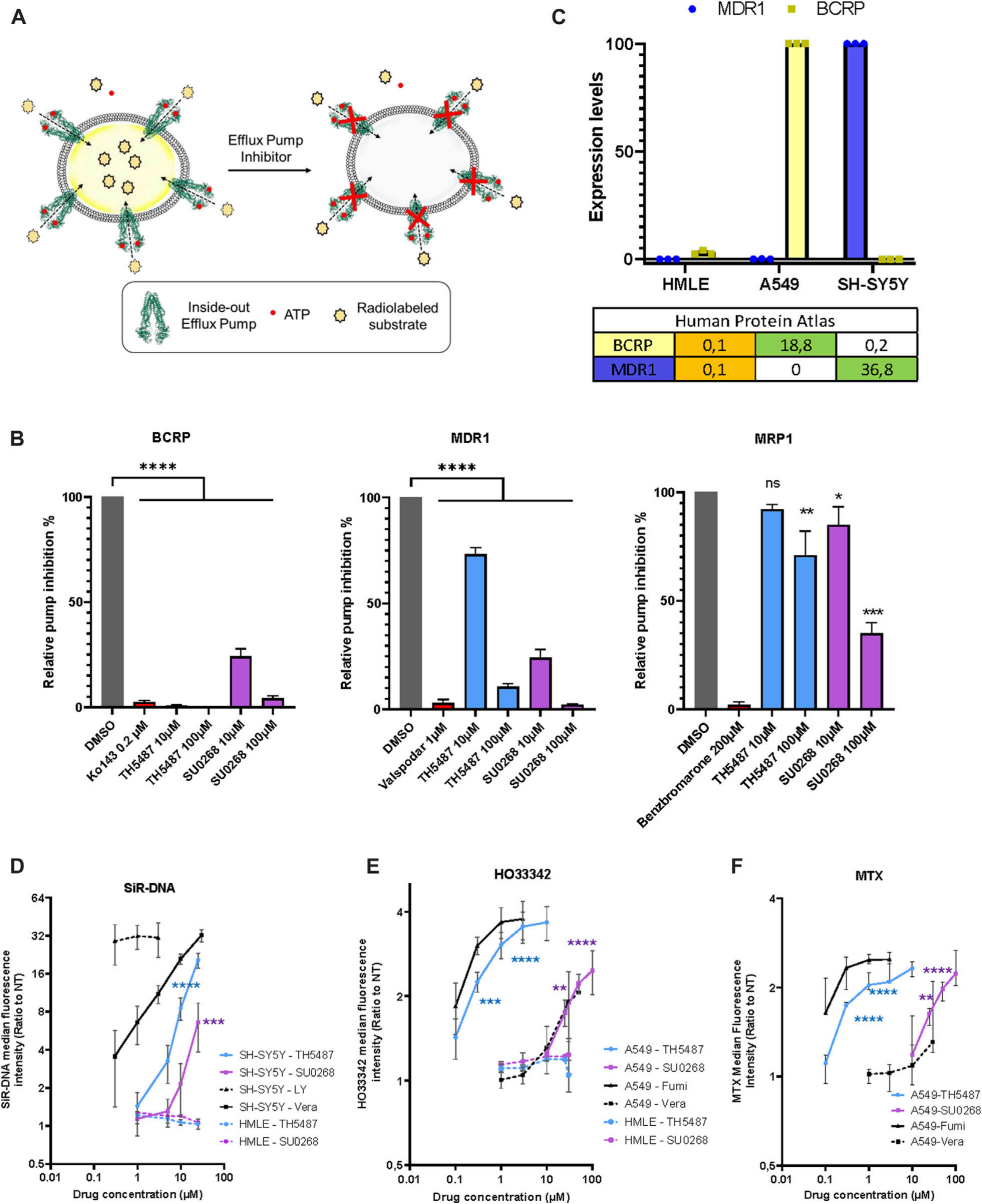
OGG1 inhibitors TH5487 and SU0268 inhibit the efflux pump activity of BCRP, MDR1 and MRP1. **(A)** Scheme illustrating the vesicle transport assay. See results and material and methods sections for details. **(B)** Vesicles expressing inside-out BCRP, MDR1 or MRP1 were incubated with the corresponding selective inhibitors (Kol143 0.2 µM—Valspodar 1 µM–Benzbromarone 200 µM), TH5487 and SU0268 (10 and 100 µM) and the specific pump substrates (E3S-NMQ-E217βG for BCRP, MDR1 or MRP1 respectively). In the graph, the substrate levels inside the vesicles are expressed as percentage of the ones in DMSO control and referred as relative pump inhibition. The means and the s.d. of three replicates are shown. The statistical analysis was performed with One-way ANOVA (*)*p* < 0.05; (**)*p* < 0.01; (***)*p* < 0.001; (****)*p* < 0.0001. **(C)** The expression of levels of BCRP and MDR1 were quantified by RT-qPCR in the indicated cell lines (HMLE, A549, and SH-SY5Y). The table reports the normalized transcript expression values as indicated in the Protein Atlas (nTPM). **(D)** SH-SY5Y and HMLE cells were stained with SiR-DNA in the presence of different concentrations of TH5487, SU0268 and the efflux pump inhibitors Verapamil (Vera) and Zosuquidar/LY-335979 (LY). **(E)** A549 and HMLE cells were stained with HO33342 in the presence of different concentrations of TH5487, SU0268 and the efflux pump inhibitors Verapamil (Vera) and Fumitremorgin C (Fumi) **(F)** A549 cells were exposed to 1 µM of Mitoxantrone (MTX) in the presence of different concentrations of TH5487, SU0268 and the efflux pump inhibitors Verapamil (Vera) and Fumitremorgin (Fumi). For **(D–F)**, the median fluorescence intensity was normalized to the median of the cells stained without inhibitors (NT) and data presented as a mean±sd for three independent experiments. Statistical analysis was performed with Ordinary one-way ANOVA (*)*p* < 0.05; (**)*p* < 0.01; (***)*p* < 0.001; (****)*p* < 0.0001.

Since the effects observed for the OGG1 inhibitors TH5487 and SU0268 were very similar to the ones observed with the general efflux pump inhibitor Verapamil, we wondered if OGG1 inhibitors could have a direct effect on efflux pumps. Many different efflux pumps have been identified in human cells, and cancer cell lines express a combination of several of them, being BCRP, MDR1, and MRP1 the most widely overexpressed ones (Wang et al., 2021; Robey et al., 2018). In order to evaluate the direct effect of OGG1 inhibitors on efflux pump activity, we used the vesicular trafficking *in vitro* assay. The principle of the assay relies on measuring the accumulation of a substrate into vesicles containing the efflux transporter in an inside out orientation, with the ATP and substrate binding sites of the transporter exposed to the buffer outside. By utilizing inside out vesicles, the ATP-dependent transport of substrate is directed into the vesicular lumen, where low permeability compounds are then essentially trapped. HEK293 cells are particularly appropriated for this kind of analysis as they express moderate levels of efflux pumps and allow to evaluate the effect of drugs on a individually overexpressed efflux pumps. Thus, vesicles were prepared from HEK293 cells overexpressing either BCRP, MRP1 or MDR1. Vesicles were incubated with the radiolabeled molecules E3S, NMQ or E_2_17β, that are specific substrates for BCRP, MDR1, and MRP1 respectively, in the presence of ATP (active transport) or AMP (background). The amount of radioactive substrate taken up into the vesicles in an ATP dependent manner, was quantified by liquid scintillation counting inside the filtered vesicles (illustration Figure 2A). Specific inhibitors for each of the evaluated pumps (Ko143 for BCRP, valspodar for MDR1, and Benzbromarone for MRP1) were used as positive controls in the assay. At the dose of 10 μM, classically used *in cellulo* experiments using OGG1 inhibitors, TH5487 showed an almost complete inhibitory effect on BCRP (99% inhibition), and a mild effect on MDR1 (27%) or MRP1 (8%). SU0268 inhibited both BCRP and MDR1 at 76% and showed a milder but still significant effect on MRP1 (15% inhibition) (Figure 2B). At a concentration of 100 μM, both OGG1 inhibitors show an inhibitory effect on the three efflux pumps analysed.

### The effect of OGG1 inhibitors is cell line dependent and correlates with the levels of expression of BCRP and MDR1

The identification of BCRP and MDR1 as direct targets of OGG1 inhibitors could explain the results presented in Figure 1 as both SiR-DNA, Mitotracker green and TMRE are known substrates of those efflux pumps (Lukinavičius et al., 2015; de Almeida et al., 2017; Mansell et al., 2021). In order to evaluate if the effects observed for OGG1 inhibitors were indeed dependent on the levels of expression of the efflux pumps we selected different cancer cell lines according to their respective expression levels of BCRP and MDR1. Based on the information available in the Human Protein Atlas (https://www.proteinatlas.org/) we decided to evaluate the effect of OGG1 inhibitors on A549 cells expressing high levels of BCRP, SH-SY5Y cells expressing high levels of MDR1, and HMLE showing very low expression of both efflux pumps. The levels of expression of BCRP and MDR1 genes were quantified in the different cellular models by RT-qPCR, and were in agreement with the data available in the Human Protein Atlas (Figure 2C). Consistently with the results obtained with the vesicular traffic assay, the effect of both TH5487 and SU0268 on the fluorescent signal obtained upon staining with SiR-DNA, a good substrate of MDR1 (Lukinavičius et al., 2015; Sen et al., 2018), was significantly stronger in SH-SY5Y cells, compared to the effects observed in HMLE cells in which MDR1 expression is undetectable (Figure 2D). We next monitored the effect of OGG1 inhibitors on fluorescent levels obtained after staining the cells with HO33342, a good substrate of BCRP (Abbott, 2003). We observed a very strong effect of the OGG1 inhibitor TH5487 on the fluorescent signal of HO33342 in A549 cells, expressing higher levels of BCRP. Interestingly, the effect of TH5487 was very similar to the observed with Fumitremorgin C, a specific and strong inhibitor of BCRP. A weaker but clearly detectable effect, was observed with the OGG1 inhibitor SU0268, that was comparable with the effect observed with the very well-known and highly used efflux pump inhibitor Verapamil (Figure 2E). These results are in agreement with those obtained in the vesicle transport assay showing that BCRP activity was inhibited at 100% by TH5487 and at 76% by SU0268. Almost no effect of TH5487 or SU0268 could be observed in HMLE cells, expressing BRCP1 at undetectable levels (Figure 2E). Altogether, these results clearly indicate a positive correlation between the level of expression of efflux pumps and the effect of OGG1 inhibitors on the accumulation of fluorescent probes inside the cells.

### Synergistic effects between OGG1 inhibitors and chemotherapeutical drugs are independent on OGG1

**FIGURE 3:**
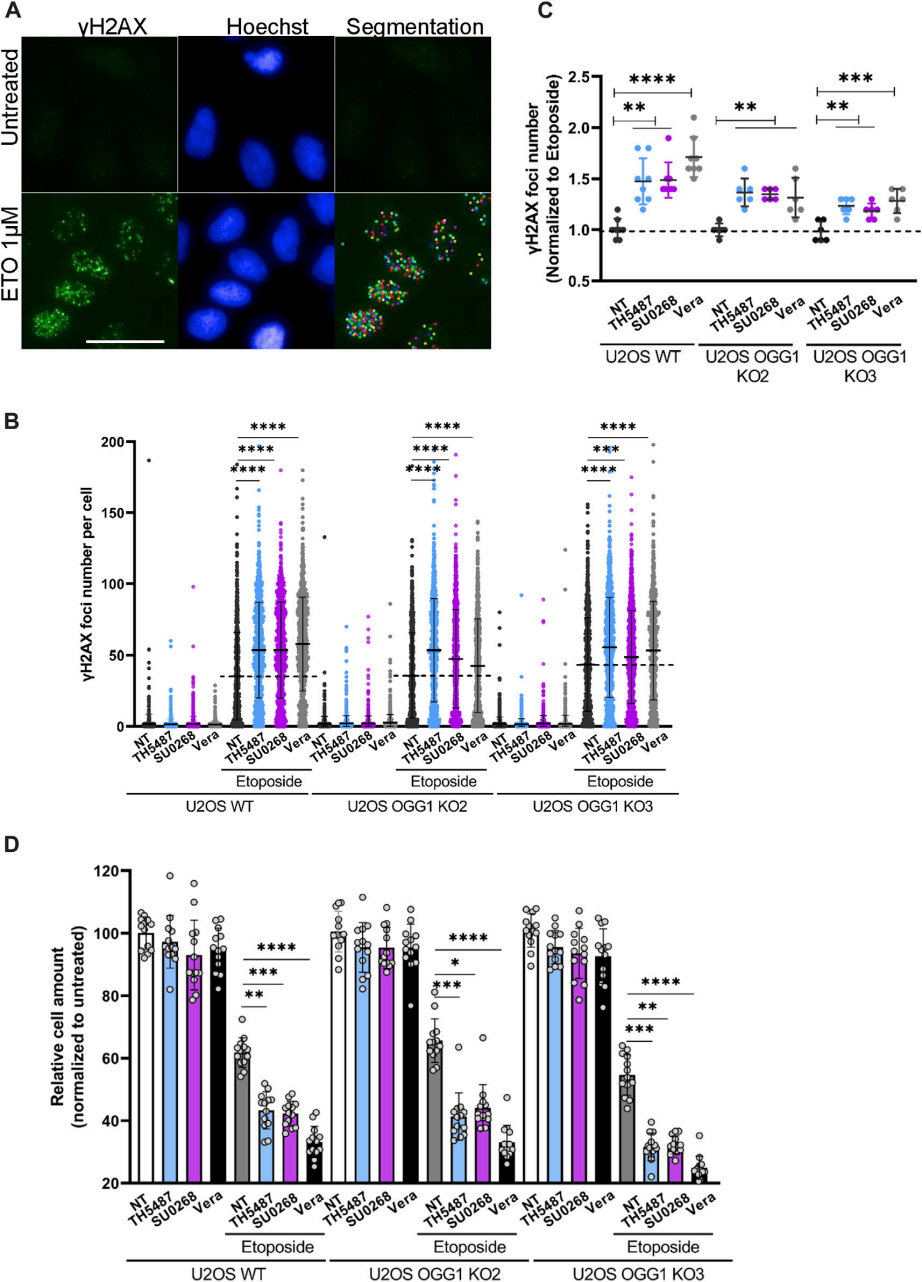
OGG1 inhibitors TH5487 and SU0268 increase the effects of the chemotherapeutical drug Etoposide, independently of OGG1. **(A)** U2OS WT and OGG1 KO cells were exposed to Etoposide alone or in combination with 10 µM of TH5487, SU0268 or Verapamil. Immediately after exposure to the drugs, cells were fixed and DSB were visualized by immunofluorescence using an antibody against γH2AX (green). Nuclei were stained with Hoechst 33342 (blue). Statistical analysis was performed with a Kruskal Wallis test. A segmentation algorithm was used to quantify γH2AX foci number in at least 1000 cells for each condition. Scale bar 50 µm. **(B)** Distribution of γH2AX foci number per nuclei is presented for a representative experiment out of three. Statistic analysis was performed with a Kruskal Wallis test (***)*p* < 0.001; (****)*p* < 0.0001. **(C)** The number of γH2AX foci per nuclei was normalized to the number of foci in cells exposed to Etoposide and the mean from three independent experiments is represented in the graph. Statistical analysis was performed with a Kruskal Wallis test (**)*p* < 0.01; (***)*p* < 0.001; (****)*p* < 0.0001. **(D)** Cell survival was evaluated in U2OS WT and OGG1 KO cells, 4 days after exposure to a single treatment with Etoposide or a combined treatment with Etoposide and 10 µM of TH5487, SU0268 or Verapamil. The number of cells were normalized to the untreated control cells. The mean of three independent experiments is represented in the graph. Statistical analysis was performed with a Kruskal Wallis test (*)*p* < 0.05; (**)*p* < 0.01; (***)*p* < 0.001; (****)*p* < 0.0001.

Not only fluorescent probes are transported by the efflux pumps but also several chemotherapeutical drugs. Indeed, many cancer cells overexpress efflux pumps allowing them to export drugs out of the cells, a phenomenon at the origin of the observed drug multiresistance in several cancers (Wang et al., 2021; Robey et al., 2018). To evaluate if OGG1 inhibitors could influence the intracellular concentration of chemotherapeutical drugs, we incubated the cells with Mitoxantrone (MTX), a Topoisomerase II inhibitor that emits a fluorescence signal and is a known substrate of BCRP (Abbott 2003). The red fluorescence emitted by MTX was measured by flow cytometry and, as observed for the other fluorescent dyes used so far in this study, higher fluorescent levels were detected in cells simultaneously incubated with MTX and the OGG1 inhibitors TH5487 and SU0268 (Figure 2F). Again, the effects of OGG1 inhibitors where comparable to the ones observed with efflux pumps inhibitors Verapamil and Fumitremorgin (Figure 2F). If the increase in the intracellular levels of MTX observed in the presence of OGG1 inhibitors is due to their inhibitory effect on efflux pumps, we could expect OGG1 inhibitors to potentiate the effect of many chemotherapeutical drugs known to be substrates of BCRP1 or MDR1 such as doxorubicin, paclitaxel, cisplatine, 5-FU, etoposide, (Wang et al., 2021). Indeed, a higher number of γH2AX foci, used as an indicator of double strand breaks (DSB), were induced by Etoposide treatment in the presence of OGG1 inhibitors TH5487 and SU0268. The fact that the same effects were observed in OGG1 deficient cells, clearly points out an off-target effect and discards the involvement of OGG1 activity (Figures 3A–C). In agreement with the higher level of DNA damage observed, cell survival was also affected, with higher mortality observed in cells treated simultaneously with Etoposide and OGG1 competitive inhibitors, compared to the ones exposed to the single treatment with Etoposide (Figure 3D). The synergistic effect observed between Etoposide and OGG1 inhibitors is independent of OGG1 activity and could be explained by the off-target effect of both TH5487 and SU0268 on efflux pumps.

### OGG1 inhibitor SU0268 perturbs mitotic progression independently on OGG1

**FIGURE 4:**
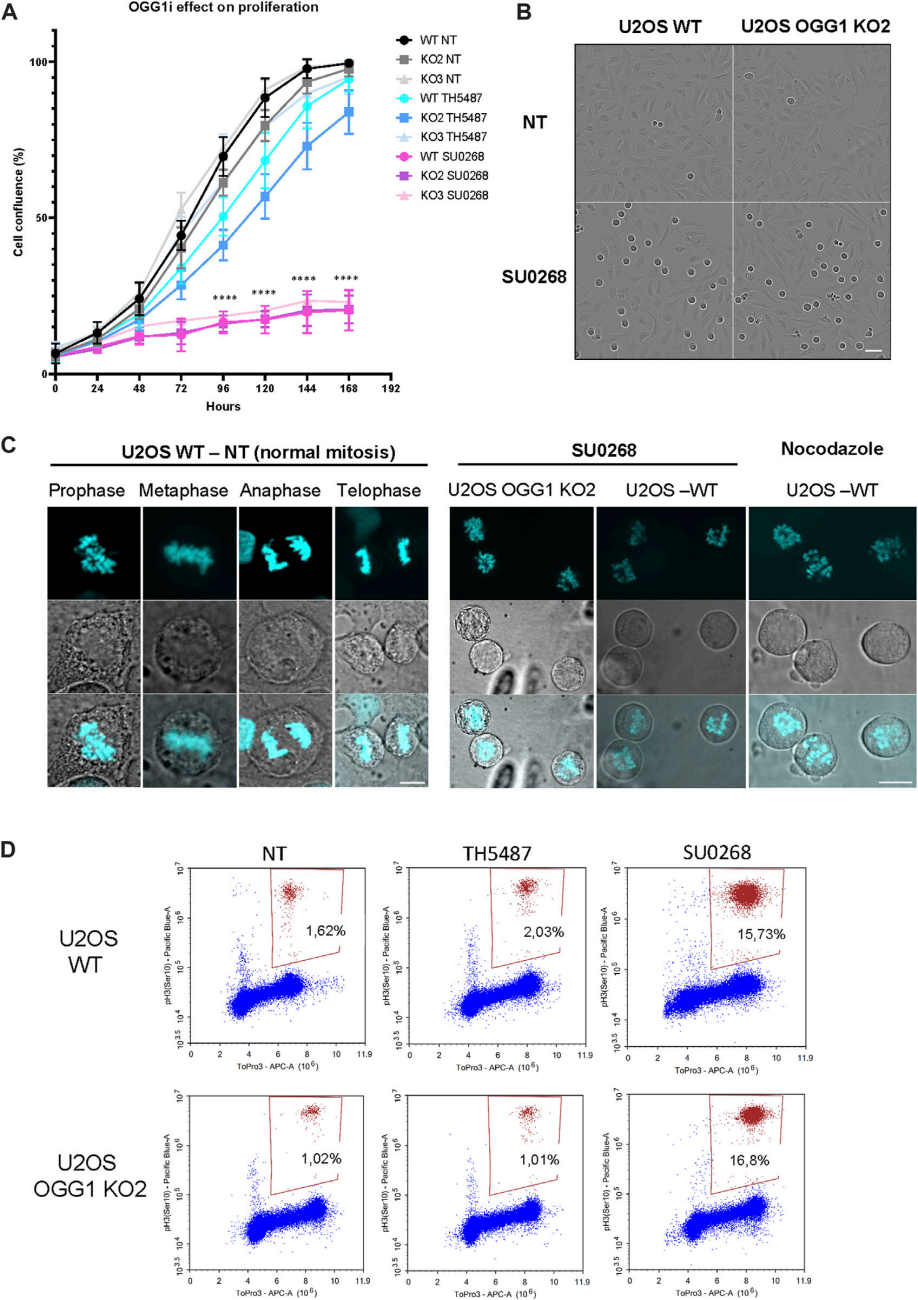
OGG1 inhibitor SU0268 impairs mitotic progression in an OGG1 independent way. **(A)** The proliferation of U2OS WT and OGG1 KO cells under chronic treatment with TH5487 and SU0268 (10 µM) was followed with Incucyte for one 1 week as described in materials and methods. Data are presented as mean confluence ±s.d. of at least three technical replicates from three independent experiments (left side). Mixed-effect analysis was performed (****)*p* < 0.0001. **(B)** Representative Incucyte transmission images of U2OS WT and OGG1 KO2 cells at basal conditions or treated with SU026. Scale bar 50 µm **(C)** Cells were exposed to Nocodazole or SU0268 for 6 h, DNA was stained with Ho33342 and cells were imaged by fluorescence microscopy. For the untreated cells, the different phases of mitosis (Prophase, Metaphase, Anaphase and Telophase) are illustrated. Only cells in Prophase were observed upon exposure to SU0268 or Nocodazole. Scale bars 10 µm. **(D)** Cells undergoing mitosis were quantified by flow cytometry using an antibody against Phospho-H3. A higher accumulation of cells in mitosis was observed for both U2OS WT and OGG1 KO2 in cells exposed to SU0268 compared to untreated cells or cells exposed to TH5487.

In order to evaluate if OGG1 inhibitors had an effect on cell proliferation and if it was dependent on OGG1, TH5487 or SU0268 were added to the culture medium at a concentration of 10 µM and cellular proliferation was monitored by using the Incucyte^®^ Live-Cell Analysis System. No significant effects could be observed in the presence of TH5487, and although cell proliferation was slightly slowed down, the effects were not dependent on OGG1 since very similar behaviour was observed between WT and OGG1 KO cells. In contrast, when 10 µM of SU0268 was added to the cell culture medium a dramatic proliferation arrest was observed for both WT and OGG1 KO cells (Figure 4A). The same effects were observed with three independent batches of SU0268 purchased from independent suppliers (see material and methods for references). The images obtained in this assay unveiled a large number of cells showing a round shape characteristic of cells undergoing mitosis (Figure 4B). Indeed, staining of genomic DNA with HO33342 revealed that both U2OS WT and OGG1 KO cells incubated in the presence of SU0268 had a defect on mitotic progression as all mitotic cells were blocked at the prophase step of the cell cycle, and no cells progressing through Metaphase, Anaphase or Telophase could be observed (Figure 4C). The mitotic figures observed in the presence of SU0268 were comparable to the ones induced by incubating the cells with Nocodazole, a molecule known to disrupt microtubule assembly/disassembly dynamics, and thus impairing formation of the metaphase spindles during the cell division cycle (Lara-Gonzalez et al., 2012). The percentage of cells undergoing mitosis was quantified by flow cytometry using an antibody specific against the mitotic marker phospho-Histone H3 (Ser10). While the level of mitotic cells was around 2% in cells grown in normal culture medium or in the presence of TH5487, up to 16% of mitotic cells accumulated 6 hours after the addition of 10 µM of SU02568 in the medium (Figure 4D). It is important to note that effects on cell cycle progression were not cell line dependent as very similar results were obtained in different cell lines such as HMLE, A549, and T47D (Supplementary Figure S3).

## Discussion

There is an increasing interest in developing inhibitors of proteins involved in DNA repair, and BER in particular, for therapeutical purposes, with many of those molecules undergoing clinical trials (Li et al., 2021; Raimundo et al., 2021). The use of BER inhibitors could impair the proliferation of cancer cells showing a high level of oxidative stress and endogenous DNA damage DNA repair (Visnes et al., 2020). Furthermore, radiotherapy and most of the chemotherapeutical drugs induce DNA lesions such as oxidation, alkylation or deamination of bases that can be repaired by the BER pathway. Therefore, the use of BER inhibitors could have a synergistic effect when used in combination with chemotherapeutical drugs (Helleday et al., 2008). Important efforts from different groups have led to the identification and characterization of inhibitors against BER proteins such as PARP1, APE1 and OGG1 (Li et al., 2021). Very promising observations were made with the OGG1 competitive inhibitors TH5487 and SU0268 that have been proposed to be potential new tools for cancer therapy (Visnes et al., 2020) and for the treatment of inflammatory diseases (Visnes et al., 2018b; Karsten, 2022). Beside the promising potential use of OGG1 inhibitors for therapeutical purposes, these molecules represent also valuable tools in fundamental research and have brought important information concerning OGG1 molecular mechanisms, in both repair and transcriptional regulation (Hanna et al., 2020; Hao et al., 2020; Qin et al., 2020; Xia et al., 2022).

Here we show that OGG1 inhibitors TH5487 and SU0268 have considerable off target effects that can affect the interpretation of the results described previously using these molecules. Our results indicate that both TH5487 and SU0268 inhibit the ATP dependent efflux pump activity of BCRP, MDR1 and to a minor extent MRP1 (Figure 2). The inhibition of the efflux pump could explain the higher intracellular accumulation of several fluorescent dyes (SiR-DNA, MitoTracker Green, TMRE, NucLight, Hoechst 33342; Figure 1, Figure 2, and Supplementary Figure S1) and chemotherapeutical drugs (mitoxantrone, Figure 2F) observed in the presence of OGG1 inhibitors. Many efflux pumps have been found to be overexpressed in cancer cells, being MDR1, BCRP and MRP1 the better characterized ones (Robey et al., 2018). The expression of several efflux pumps has been associated with the development of drug resistance and it is well documented that the inhibition of efflux pumps, by increasing the concentration of drugs inside the cells, increase cellular sensitivity. A wide range of anticancer drugs have been identified as substrates of BCRP or MDR1, including chemotherapeutic agents such as doxorubicin, paclitaxel, vincristine, tyrosine kinase inhibitors, cisplatine, 5-FU and etoposide (Wang et al., 2021). Therefore, the synergistic effects observed between etoposide and OGG1 inhibitors on the number of γH2AX foci and cellular sensitivity could be explained by the higher intracellular accumulation of the drug. Since the same effects were observed in OGG1 KO cells, we conclude that they were related to an off-target effect and not to the inhibition of OGG1 activity (Figure 3).

It has previously been proposed that inhibition of OGG1 by TH5487 could potentiate methotrexate anticancer effects by blocking oxidative DNA damage at telomeres leading to the induction of DSB (Baquero et al., 2021). This statement was based on the observation of a very high number of 53BP foci in cells exposed to a combined treatment with methotrexate and TH5487, compared to the cells treated with methotrexate alone. However, considering our observations, an alternative explanation could be that TH5487 induces a higher intracellular concentration of methotrexate through the inhibition of efflux pumps. In apparent contradiction with these results, reduced levels of γH2AX foci were detected in U2OS cells exposed to menadione or KBrO_3_ in combination with TH5487, leading the authors to conclude that this was due to a reduction in incisions (SSB) generated as intermediates of BER in the absence of OGG1 activity (Hanna et al., 2020). Thus, from a molecular point of view, it remains to be established whether deficiency in OGG1 activity results in higher (Baquero et al., 2021) or lower (Wang et al., 2018; Hanna et al., 2020) levels of DSB.

Several studies suggested that deficiency or inhibition of OGG1 limits the formation of SSB and thus increases resistance to PARP inhibitors (Giovannini et al., 2019). However, several reports have shown that the absence of OGG1 results in a higher sensitivity to PARP inhibitors (Alli et al., 2009; Noren-Hooten et al., 2011). In agreement with these reports, TH5487 was shown to enhance the sensitivity to the PARP inhibitor Olaparib, in particular in the context of BRCA1 deficiency (Baquero et al., 2022). This could represent a potential interesting strategy to overcome resistance to PARP inhibitors (Mweempwa and Wilson, 2019). However, the effects of TH5487 were not evaluated in OGG1 deficient cells, making it difficult to assess if they were really due to inhibition of the DNA glycosylase activity or to an off-target effect. The role of efflux pumps in the resistance to PARPi has been documented in several human colorectal cancer cell lines. Co-treatment of PARPi with the efflux pump inhibitor Verapamil increases the intracellular concentration of the inhibitor and the cellular sensitivity (Oplustilova et al., 2012). Long-term exposure to PARP inhibitor Olaparib has been shown to induce the overexpression of MDR1 and BCRP1, leading to drug resistance in mouse mammary tumours (Rottenberg et al., 2008), that could be reversed by pharmacological inhibition or deletion of MDR1 (Jaspers et al., 2013). Considering that PARP inhibitors are substrates of BCRP1 and MDR1 (Durmus et al., 2015) and that these two efflux pumps are dramatically inhibited by TH5487 (Figure 2), it cannot be excluded that the synergistic effects observed between the two inhibitors could indeed be due to a higher intracellular accumulation of PARPi in the presence of TH5487.

Therefore the synergistic effects described between OGG1i and other small molecules (such as chemotherapeutical drugs or PARP inhibitors) should be carefully re-evaluated. Many controls need to be included in the experimental settings, and imperatively OGG1 deficient cells, in order to evaluate if the observed effects are related to the inactivation of the protein or could be explained by off-target effects. As shown in Figure 2 the level of expression of efflux pumps is extremely heterogeneous in different cell lines and could therefore affect the interpretation of the results obtained using the OGG1 inhibitors in the different experimental models.

In addition to the effect of OGG1i on the activity of efflux pumps, we have also observed an effect of SU0268 on mitotic progression. Interestingly, a similar off-target effect has been reported for one of the first inhibitors described for MTH1 (Gad et al., 2014), a protein that sanitizes the pool of oxidized nucleotides and whose inhibition results in the accumulation of genomic 8-oxoG (Berglund et al., 2016). However, the effect of MTH1 inhibitor TH588 on mitotic progression leading to cancer cell proliferation arrest were shown to be independent on MTH1 but mostly due to an off-effect of the molecule as a microtubule-modulating agent (Gul et al., 2019). Indeed, several highly selective and potent inhibitors of MTH1 have failed to show any cytotoxicity (Kawamura et al., 2016; Kettle et al., 2016; Petrocchi et al., 2016), arguing against a specific effect of MTH1 inhibition being at the origin of cancer cell death. Altogether, it remains unclear if the accumulation of 8-oxoG induced by several MTH1 inhibitors is related to mitotic progression arrest and cancer cell death (Moukengue et al., 2020; Rudd et al., 2020; Sanjiv et al., 2021). However, the effect of SU0268 on mitotic progression observed in this study is probably not related to the accumulation of 8-oxoG as no major effects on the cell cycle progression were observed for TH5487 that also efficiently impairs the repair of 8-oxoG (Supplementary Figures S2,S3). Furthermore, the same mitotic arrest was observed in WT cells and in cells deficient for OGG1, indicating that this response was due to an off-target effect, independent of OGG1 activity or the accumulation of 8-oxoG.

Even if combined therapies using different inhibitors or dual-target inhibitors have proven their efficacy in some cases (Chen et al., 2022), it is not possible to predict the potential benefit of these dual effects for the treatment of particular patients. In view of the results presented here, it could be tempting to speculate that the inhibitory effect of SU0268 and TH5487 on both the DNA repair protein OGG1 and efflux pumps could have a synergistic effect and improve the efficacy of chemotherapeutical drugs. However, those short-cuts are often dangerous as it is difficult to quantify the abundance and heterogeneity of ABC transporters in particular tumours and we have no definitive proof that inhibitors increase drug accumulation in cancer cells without unacceptable toxicity. Indeed, despite the significant effort over the last decades to develop efflux pump inhibitors able to counteract multidrug resistance, no promising molecules reached clinical application due to toxicity and poor response (Wang et al., 2021). The toxicity of those molecules can be explained in part by the pleiotropic expression of the efflux pumps and their major physiological roles in avoiding the transport of exogenous molecules across the blood brain barrier (BBB), the blood-testis, and blood-placental barriers.

Off-target effects of small molecule drugs is at the origin of severe side effects and is a major cause of clinical trials failure (Sun et al., 2022). A systematic knock-out of the proteins targeted by several drugs already in pre-clinical or clinical studies, has unveiled that in most of the cases the effect of the drug was unaffected by the loss of its putative target, indicating that the cytotoxicity at the origin of cancer cell death was due to off-target effects (Lin et al., 2019). Thus, it is extremely important to characterize the molecular mechanisms of action and the potential off-target effects of these drugs in order to reduce the number of “failed” clinical studies and improve the success rate of the molecule to reach the clinics and prove its benefits for human.

**FIGURE 5:**
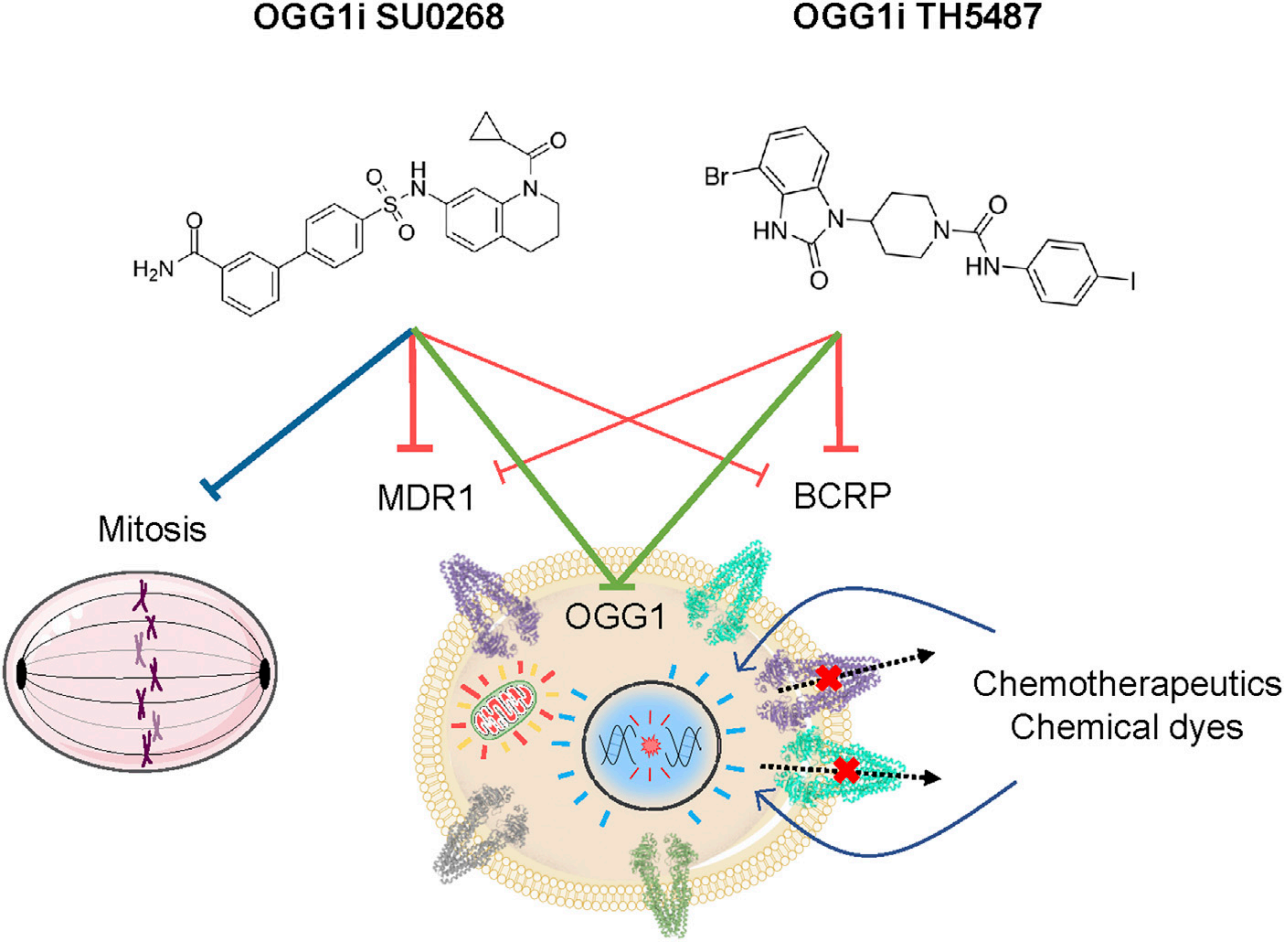
Cellular effects of the OGG1 inhibitors TH5487 and SU0268. Beside their activity as inhibitors of the DNA glycosylase activity of OGG1, TH5487 and SU0268 have several off-target effects that are independent on OGG1. Both molecules inhibit the activity of the efflux pumps MDR1 and BCRP1, thus increasing the intracellular concentration of fluorescent dyes and chemotherapeutical molecules. In addition, SU0268 impairs mitotic progression.

There is a major interest on OGG1 inhibitors, not only for their potential therapeutical use but also from a fundamental point of view as they could provide a better understanding on the role of OGG1 In both DNA repair and transcriptional regulation. Our study unveils two off-target effects of OGG1 competitive inhibitors, TH5487 and SU0268, on efflux pumps and mitotic progression, both independent on OGG1 activity (Figure 5). Therefore, a better characterization of the molecular mechanism of action of these molecules, and the development of optimized more specific molecules, are essential.

## Data Availability

The original contributions presented in the study are included in the article/Supplementary Material, further inquiries can be directed to the corresponding author.

## Supplementary material

Supplementary Figure S1 The addition of OGG1 competitive inhibitors TH5487 and SU0268 to the medium results in higher staining with the fluorescent dye NucLight. Representative images obtained with the high-content imaging microscope used for the quantifications presented in Figure 1D. DNA was stained with both Picogreen (green) and NucLight (red) in untreated cells or cells exposed to TH5487, SU0268 or Verapamil at 10 or 50 µM. Scale bar 200 µm.

Supplementary Figure S2 8-oxoG repair kinetics is impaired in the presence of OGG1 inhibitors TH5487 and SU0268. **(A)** HeLa cells overexpressing OGG1-GFP were exposed to 40 mM of KBrO3 for 30 minutes. After the treatment cells were washed and incubated for 4 hours in DMEM in order to allow the repair of the induced lesions. 8-oxoG (green) was quantified by immunofluorescence using an antibody against the lesion. An antibody against GFP was used to visualise cells overexpressing OGG1-GFP (magenta). **(B)** cells overexpressing OGG1-GFP repair 8-oxoG with a faster dynamics compared to HeLa cells expressing the endogenous levels of OGG1. Statistical analysis was performed with Kruskal-Wallis test (****)P<.0001. **(C)** 8-oxoG levels remaining 4 h after exposure to KBrO3 were quantified in cells overexpressing OGG1-GFP exposed or not to OGG1 inhibitors TH5487 and SU0268. Cells transfected with an siRNA against OGG1 were used as a control. At least 1000 cells were analysed for each condition and statistical analysis was as performed with a Kruskal Wallis test (****)P<.0001. One representative experiment out of three is presented. **(D)** Correlation analysis between the OGG1-GFP expression and the remaining 8-oxoG 4 h after exposure to KBrO3 in cells exposed or not to OGG1 inhibitors TH5487 and SU0268.

Supplementary Figure S3 SU0268 induces mitosis block in different cell lines. Cells undergoing mitosis were quantified by flow cytometry using an antibody against Phospho-H3. A higher accumulation of cells in mitosis was observed for HMLE, T47D, and A549 cells exposed to SU0268 compared to untreated cells or cells exposed to TH5487. The percentage of cells positive for phosphor-H3 is indicated in the graph.
