# Supplementary material for: OGG1 competitive inhibitors show important off-target effects by directly inhibiting efflux pumps and disturbing mitotic progression
Source: Front Cell Dev Biol. 2023 Feb 3;11:1124960. doi: 10.3389/fcell.2023.1124960 (PMC9936318; doi:10.3389/fcell.2023.1124960)
Supplement: Supplementary file 1 [file DataSheet1.pdf]

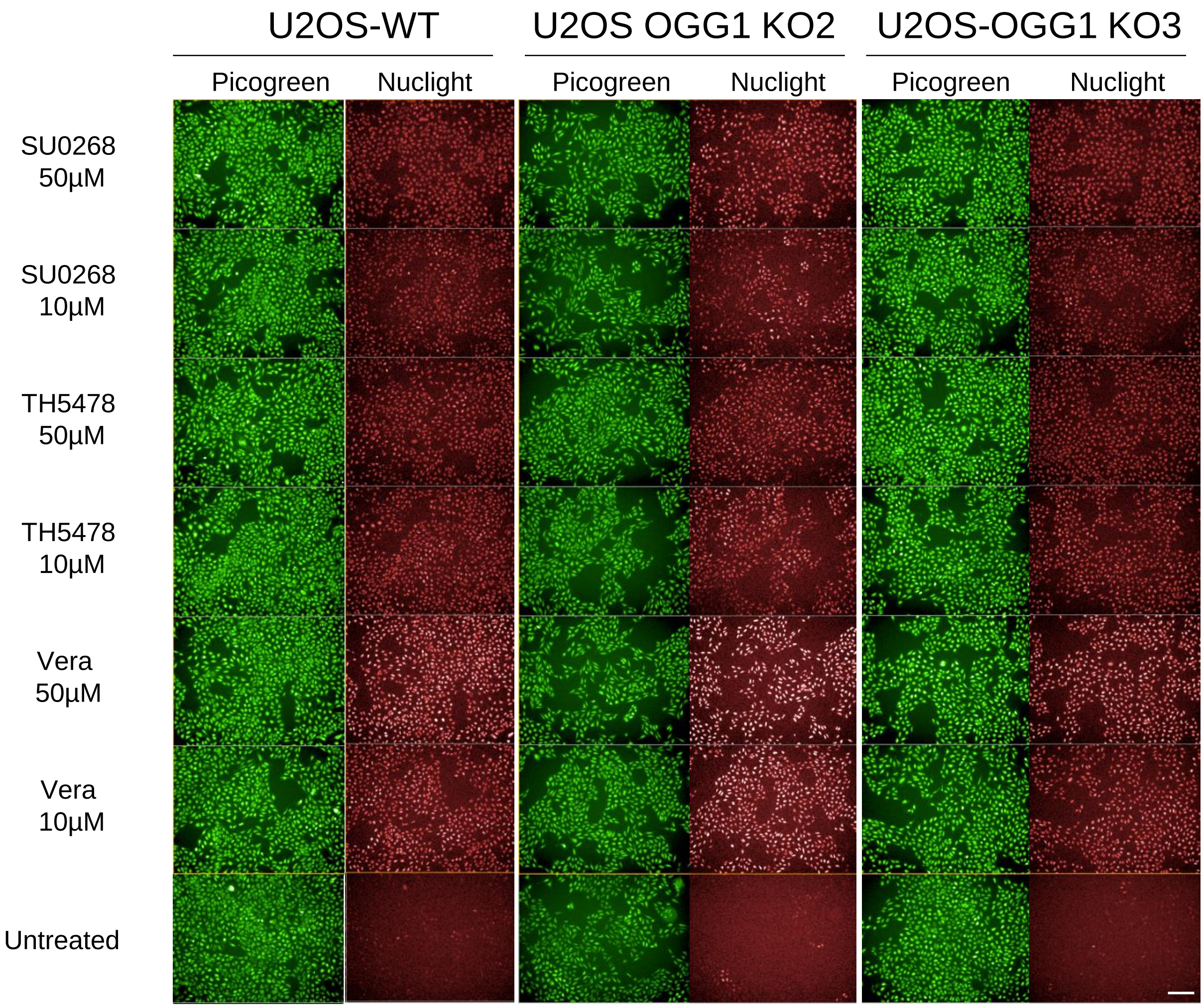

<u>Figure S1.</u> The addition of OGG1 competitive inhibitors TH5487 and SU0268 to the medium results in higher staining with the fluorescent dye NucLight. Representative images obtained with the high-content imaging microscope used for the quantifications presented in Figure 1D. DNA was stained with both Picogreen (green) and NucLight (red) in untreated cells or cells exposed to TH5487, SU0268 or Verapamil at 10 or 50 µM. Scale bar 200 µm

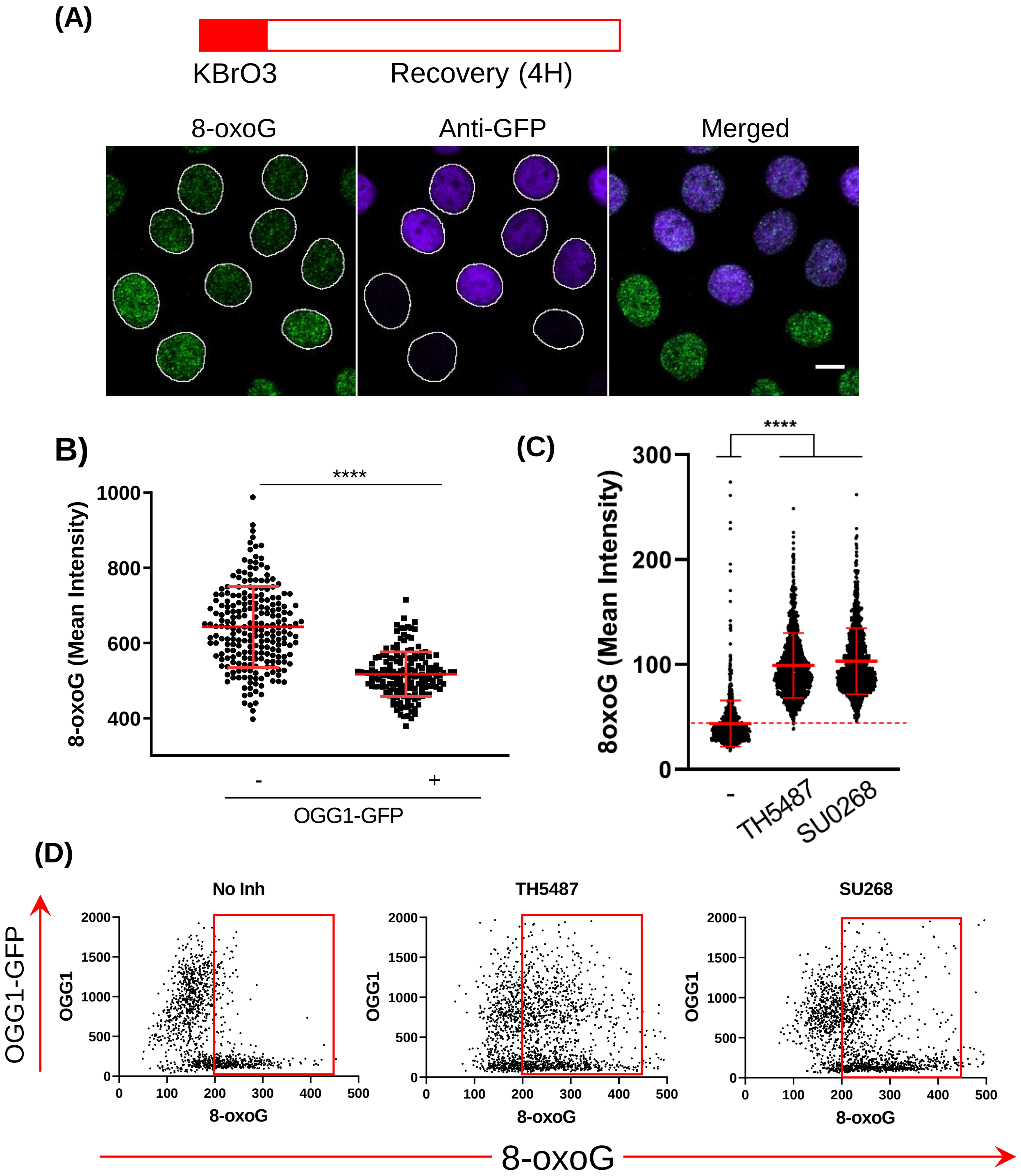

Figure S2. 8-oxoG repair kinetics is impaired in the presence of OGG1 inhibitors TH5487 and SU0268. A) HeLa cells overexpressing OGG1-GFP were exposed to 40 mM of KBrO3 for 30 minutes. After the treatment cells were washed and incubated for 4 hours in DMEM in order to allow the repair of the induced lesions. 8-oxoG (green) was quantified by immunofluorescence using an antibody against the lesion. An antibody against GFP was used to visualise cells overexpressing OGG1-GFP (magenta). (B) cells overexpressing OGG1-GFP repair 8-oxoG with a faster dynamics compared to HeLa cells expressing the endogenous levels of OGG1. Statistical analysis was performed with Kruskal-Wallis test (****)P<0.0001. (C) 8-oxoG levels remaining 4 hours after exposure to KBrO3 were quantified in cells overexpressing OGG1-GFP exposed or not to OGG1 inhibitors TH5487 and SU0268. Cells transfected with an siRNA against OGG1 were used as a control. At least 1000 cells were analysed for each condition and statistical analysis was as performed with a Kruskal Wallis test (****)P<0.0001. One representative experiment out of three is presented. (D) Correlation analysis between the OGG1-GFP expression and the remaining 8-oxoG 4 hours after exposure to KBrO3 in cells exposed or not to OGG1 inhibitors TH5487 and SU0268.

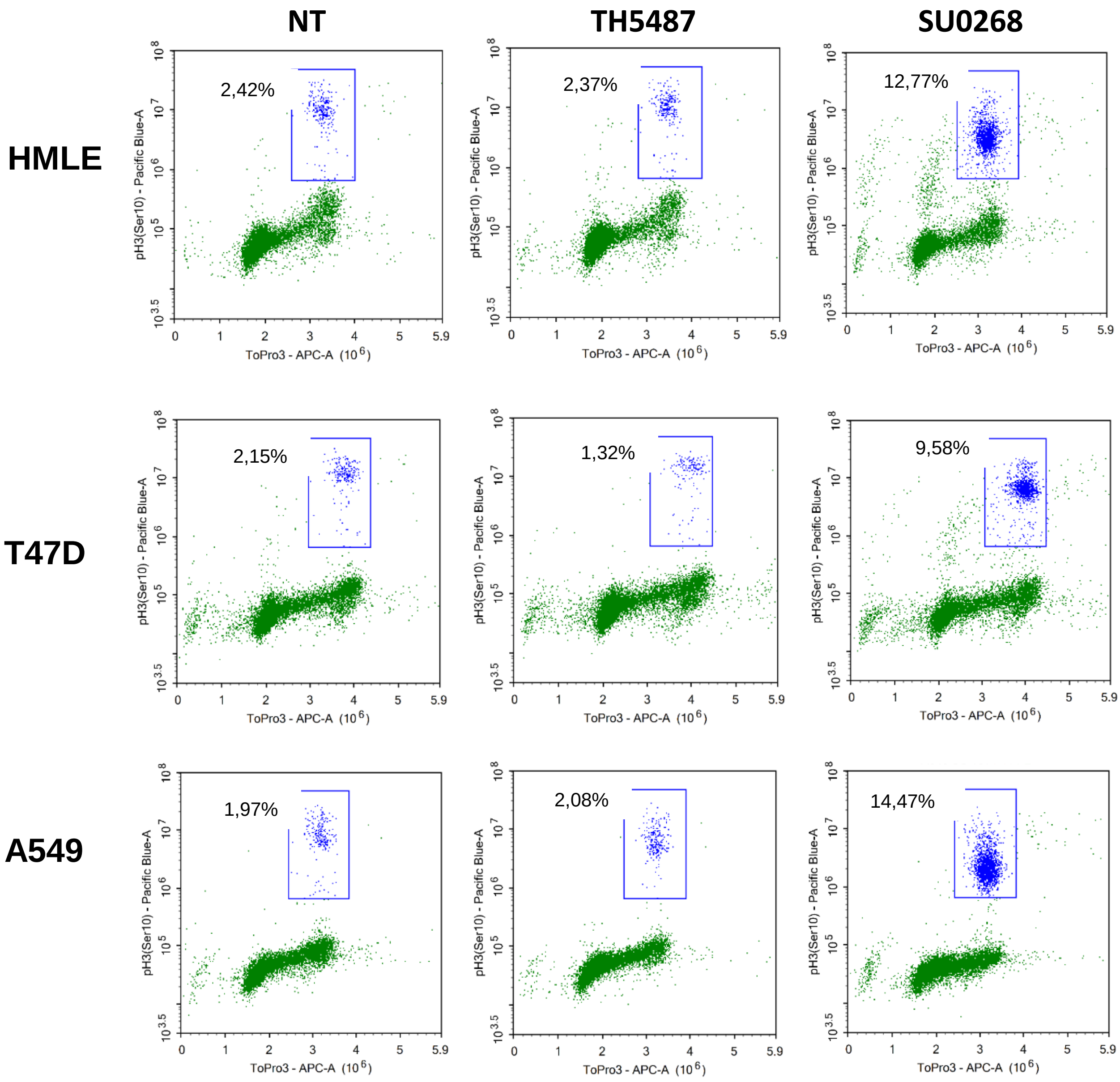

Figure S3. SU0268 induces mitosis block in different cell lines. Cells undergoing mitosis were quantified by flow cytometry using an antibody against Phospho-H3. A higher accumulation of cells in mitosis was observed for HMLE, T47D and A549 cells exposed to SU0268 compared to untreated cells or cells exposed to TH5487. The percentage of cells positive for phosphor-H3 is indicated in the graph.
